# Combination of multi-modal MRI radiomics and liquid biopsy technique for preoperatively non-invasive diagnosis of glioma based on deep learning: protocol for a double-center, ambispective, diagnostical observational study

**DOI:** 10.3389/fnmol.2023.1183032

**Published:** 2023-05-02

**Authors:** Ping Hu, Ling Xu, Yangzhi Qi, Tengfeng Yan, Liguo Ye, Shen Wen, Dalong Yuan, Xinyi Zhu, Shuhang Deng, Xun Liu, Panpan Xu, Ran You, Dongfang Wang, Shanwen Liang, Yu Wu, Yang Xu, Qian Sun, Senlin Du, Ye Yuan, Gang Deng, Jing Cheng, Dong Zhang, Qianxue Chen, Xingen Zhu

**Affiliations:** ^1^Department of Neurosurgery, The Second Affiliated Hospital of Nanchang University, Nanchang, Jiangxi, China; ^2^Department of Neurosurgery, Renmin Hospital of Wuhan University, Wuhan, Hubei, China; ^3^School of Physics and Technology, Wuhan University, Wuhan, Hubei, China; ^4^Department of Neurosurgery, Peking Union Medical College Hospital, Chinese Academy of Medical Sciences and Peking Union Medical College, Beijing, China

**Keywords:** glioma, radiomic, liquid biopsy, circulating tumor cell, histopathology, molecular pathology, diagnosis

## Abstract

**Background:**

2021 World Health Organization (WHO) Central Nervous System (CNS) tumor classification increasingly emphasizes the important role of molecular markers in glioma diagnoses. Preoperatively non-invasive “integrated diagnosis” will bring great benefits to the treatment and prognosis of these patients with special tumor locations that cannot receive craniotomy or needle biopsy. Magnetic resonance imaging (MRI) radiomics and liquid biopsy (LB) have great potential for non-invasive diagnosis of molecular markers and grading since they are both easy to perform. This study aims to build a novel multi-task deep learning (DL) radiomic model to achieve preoperative non-invasive “integrated diagnosis” of glioma based on the 2021 WHO-CNS classification and explore whether the DL model with LB parameters can improve the performance of glioma diagnosis.

**Methods:**

This is a double-center, ambispective, diagnostical observational study. One public database named the 2019 Brain Tumor Segmentation challenge dataset (BraTS) and two original datasets, including the Second Affiliated Hospital of Nanchang University, and Renmin Hospital of Wuhan University, will be used to develop the multi-task DL radiomic model. As one of the LB techniques, circulating tumor cell (CTC) parameters will be additionally applied in the DL radiomic model for assisting the “integrated diagnosis” of glioma. The segmentation model will be evaluated with the Dice index, and the performance of the DL model for WHO grading and all molecular subtype will be evaluated with the indicators of accuracy, precision, and recall.

**Discussion:**

Simply relying on radiomics features to find the correlation with the molecular subtypes of gliomas can no longer meet the need for “precisely integrated prediction.” CTC features are a promising biomarker that may provide new directions in the exploration of “precision integrated prediction” based on the radiomics, and this is the first original study that combination of radiomics and LB technology for glioma diagnosis. We firmly believe that this innovative work will surely lay a good foundation for the “precisely integrated prediction” of glioma and point out further directions for future research.

**Clinical trail registration:**

This study was registered on ClinicalTrails.gov on 09/10/2022 with Identifier NCT05536024.

## Background

**Figure 1:**
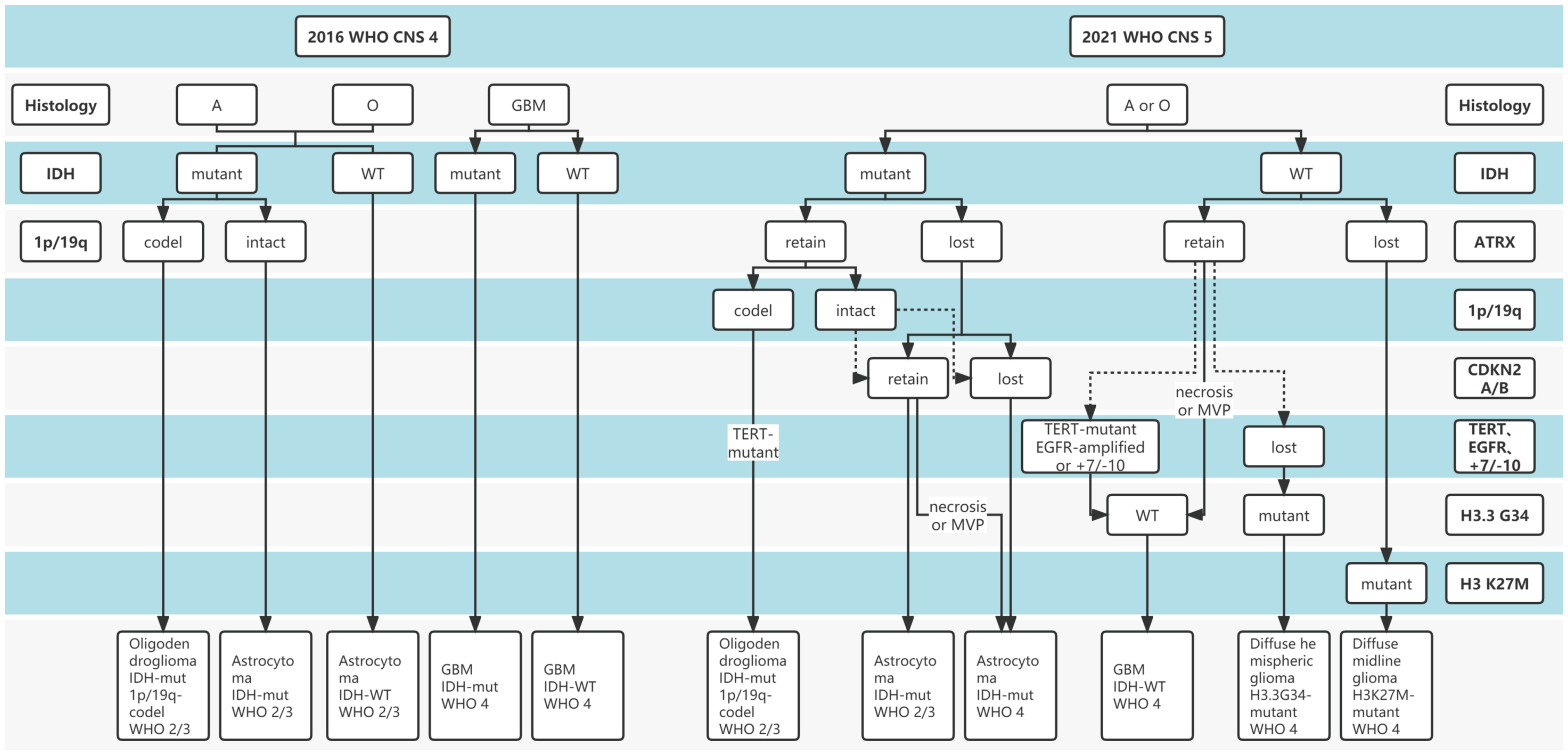
The distinction between 2016 and 2021 World Health Organization (WHO) Central Nervous System (CNS) tumor classification.

Gliomas are the most common primary intracranial malignancies, accounting for 27% of all primary brain tumors, and ~100,000 people are diagnosed with diffuse gliomas worldwide each year (Ostrom et al., 2014). To date, “integrated diagnosis” was considered the gold standard for glioma diagnosis, which combines histopathology, molecular pathology, and World Health Organization (WHO) grade (Balana et al., 2022). Previous glioma diagnostic criteria have primarily relied on histopathological biopsies, while histological classification has traditionally been determined based on tumor morphology, resulting in intra-observer variability due to intra-tumor spatial heterogeneity and sampling errors (van den Bent, 2010; Jin et al., 2021; Wijethilake et al., 2021). In addition, Traditional histopathology is somewhat difficult to explain why patients with the same pathology have significantly different survival. Over the past decade, advances in molecular pathology and histopathology detection techniques have deepened our understanding of the molecular features and biology of gliomas (Ferris et al., 2017; Acs et al., 2020). Increasing evidence revealed the important role of molecular status in the “integrated diagnosis” of glioma (Weller et al., 2021; Gritsch et al., 2022; Horbinski et al., 2022). In particular, after the concept of molecular diagnosis was proposed by the 2016 WHO Central Nervous System (CNS) classification, the 2021 CNS classification (CNS5) reemphasized the importance of molecular biomarkers in gliomas diagnosis and treatment guidelines (Figure 1), including isocitrate dehydrogenase gene (IDH) mutation status, alpha-thalassemia/mental retardation syndrome X-linked (ATRX) deletion status, 1p19q deletion status, etc. (Louis et al., 2021; Weller et al., 2021). The objective is to classify the tumor subtypes more systematically and categorize the glioma patients with similar efficacy and prognosis into a subgroup.

The current standard of therapy for gliomas is surgical resection followed by radiotherapy and/or chemotherapy based on clinical and tumor grade and molecular characteristics (Rudà et al., 2018; Weller et al., 2021). Preoperatively non-invasive and accurate early “integrated diagnosis” will bring great benefits to the treatment and prognosis of patients, especially for those with special tumor locations that cannot receive craniotomy or needle biopsy. Such special patients can take experimental therapy based on non-invasively diagnostic results. Although diagnostic criteria for molecular information are often based on tissue biopsy, other techniques, such as radiomics, radiogenomics, and liquid biopsy, have shown promise (Seow et al., 2018; Le Rhun et al., 2020; Müller Bark et al., 2020; Sareen et al., 2020; Jain and Chi, 2021; Sanvito et al., 2021; Balana et al., 2022). At present, conventional magnetic resonance imaging (MRI) scans are still the main method to assist in the diagnosis of gliomas, including pre-and post-contrast T1w, T2w, and T2w-FLAIR. Multimodal radiomics based on deep learning (DL) can comprehensively reflect the growth and morphological characteristics of gliomas, to conduct “integrated prediction” of gliomas (Gore et al., 2021).

## Related study

In recent studies, van der Voort et al. (2022) collected 1,508 patients with glioma from 16 institutions, they utilized the preoperative MRI scans to develop the multi-task conventional neural network (CNN) model and achieved the area under the receiver operator characteristic curve of the WHO grade (II/III/IV) with 0.81, IDH mutation status with 0.9, and 1p19q co-deletion status with 0.85 in the test set. Although the proposed model by R van der Voort et al. demonstrated high performance in glioma prediction, the relatively small number of molecular labels may limit the application of the model according to CNS5. The best DL model developed by Matsui et al. achieved an overall accuracy of 65.9% in predicting IDH mutation and 1p/19q co-deletion in 217 low-grade glioma patients (Matsui et al., 2020). Also, the multi-task CNN model constructed by Decuyper et al. achieved an accuracy of 94, 86, and 87% in predicting grades, IDH mutations, and 1p/19q co-deletion states in the external validation dataset with 110 patients (Decuyper et al., 2021). The model constructed by Luo et al. achieved 83.9 and 80.4% in external tests for histological and molecular subtype diagnosis (Luo et al., 2021).

In addition, there are many studies aimed to construct a non-invasive diagnostic model to predict WHO grade or single molecular markers based on the 2016 CNS classification (van der Voort et al., 2019; Bangalore Yogananda et al., 2020; Wijethilake et al., 2020; Casale et al., 2021; Choi et al., 2021; Pei et al., 2021), which is far from the comprehensive diagnosis concept of glioma and cannot meet the actual needs of non-invasive diagnosis of glioma under the constantly updated treatment concept. Therefore, a multi-task DL radiomics model for preoperatively and non-invasively predicting glioma grading and more significant molecular markers is urgently needed according to the latest 2021 CNS classification.

Radiomics has shown some feasibility in predicting tumor molecular pathology, it is ridiculous to administer precision-targeted therapy solely based on this prediction. Therefore, we hope to provide more clinical evidence for the molecular pathological diagnosis of glioma patients by using the liquid biopsy (LB) technique as an important complement to radiomics. Circulating tumor cells (CTC) is an important LB technique for tumor diagnosis, with the advantages of being non-invasive and accurate. Our previous study showed that CTC was an effective diagnostic marker for gliomas (Qi et al., 2021). Despite that no correlation was observed between CTC level and tumor histopathology, we found CTC closely related to IDH mutation status. Through the single-cell sequencing technique exemplified by multiple annealing and looping-based amplification cycles (MALBAC), researchers can obtain molecular pathology from CTC rather than tissue. These findings strongly supported that CTC can be an important complement to radiomics in glioma diagnosis. However, it must be noted that there is no common criterion for the detection and sequencing of gliomas CTC.

Based on the several limitations of the current diagnostic models of glioma, the combined methods of radiomics and LB have great potential to non-invasively diagnose glioma grading and molecular markers since they are both easy to perform. Furthermore, to our knowledge, there is no study for preoperatively non-invasive diagnosis of glioma in the context of LB-assisted radiomics.

Therefore, this study has the following objectives. First, according to the guidelines of the 2021 WHO-CNS classification, we will develop a multi-task DL model for simultaneous diagnosis of glioma classification (WHO II/III/IV) and all molecular subtypes, including IDH mutation, ATRX deletion status, 1p19q co-deletion, etc. Second, based on the same ultimate purpose of LB and radiomics, we innovatively put forward the concept and idea of combining LB technology and radiomics to investigate the performance of glioma diagnosis, and we hypothesize that adding CTC into the multitask DL model would improve the integrated diagnostic performance of glioma. This work will provide some clinical validation for this concept, hoping to supply some new ideas for subsequent research and support clinical decision-making.

## Methods

### Study design

The study design is a double-center, ambispective, diagnostical observational study, which enrolls glioma patients aged over 18 years. All eligible glioma patients will be recruited. Preoperative peripheral venous blood and postoperative tumor tissue samples will be collected to detect CTC characteristics and the latest glioma diagnosis based on the 2021 WHO-CNS tumor classification.

### Participant criterion

In this study, glioma patients from the Second Affiliated Hospital of Nanchang University, Renmin Hospital of Wuhan University, and one public database named the 2019 Brain Tumor Segmentation challenge dataset (BraTS) will be enrolled.

The inclusion criteria are as follows: (1) primary glioma; (2) aged over 18 years; (3) receiving surgical resection or needle biopsy for the first time; (4) without any radiotherapy and/or chemotherapy before a preoperative MRI scan.

The exclusion criteria are as follows: (1) secondary glioma; (2) undergone surgical treatment at admission; (3) missing MRI scan.

### Data collection

We will collect baseline characteristics from glioma patients, including age, sex, preoperative MRI datasets (T1C, T1, T2, T2 flair), CTC count, single-cell sequencing results of CTC, WHO grade, IDH, 1p/19q, ATRX, CDKN2A/B, telomerase reverse transcriptase (TERT), epidermal growth factor receptor (EGFR), +7/−10, H3.3 G34, H3K27M.

### Outcomes

#### Primary outcomes

The molecular type of 2021 WHO-CNS classification will be the primary outcome, including IDH (mutant/wild), ATRX (retain/lost), 1p/19q (codel/intact), CDKN2A/B (retain/lost), TERT/EGRF/+7/−10 (retain/lost), H3.3 G34 (mutant/wild), H3K27M mutant.

#### Secondary outcomes

WHO II/III/IV will be the secondary outcome.

### Sample size

A sample of 400 achieves 90% power to detect a difference of 0.1000 between the area under the ROC curve (AUC) under the null hypothesis of 0.7000 and an AUC under the alternative hypothesis of 0.8000 using a two-sided z-test at a significance level of 0.05000. The data are discrete (rating scale) responses. The AUC is computed between false positive rates of 0.000 and 1.000. The ratio of the standard deviation of the responses in the negative group to the standard deviation of the responses in the positive group is 1.000.

### Liquid biopsy technique

#### Collection of peripheral blood

After receiving written informed consent, peripheral blood collections were obtained from patients under Institutional Review Board-approved protocols. All patients in the study were free of significant comorbid medical conditions or prior cancer, deemed operable, and underwent a biopsy, subtotal, or gross total, surgical resection. Peripheral blood samples (5 mL × 2) were collected in EDTA buffer and processed by the device through the automatic isolation and staining procedure.

#### CTC detection

The blood sample (5 ml) was diluted 1:2 with BD wash buffer (BD, USA) containing 0.2% paraformaldehyde (PFA), 0.1% bovine serum albumin (BSA), and 0.0372% EDTA. It was incubated for 10 min at room temperature and then detected by the device. The filtrate was gently aspirated by a vacuum suction pump. After aspiration, the retained cells were washed three times with pure water and fixed in 100% methanol. After disassembly from the filter, the membrane was placed on a slide and coverslipped after it had air-dried.

The slides were immersed in 100% xylene for several minutes at room temperature until the cover glasses dropped off. Then, we added eosin to the membrane for 2 min and then discarded it. Next, we added methylene blue for 1 min and then washed it with PBS. Then, the membrane was air-dried and observed by light microscopy (Qi et al., 2021; Zhu et al., 2022). The criteria for the identification of CTCs and CTC clusters used the cytomorphological criteria proposed by other research groups. The results of Wright’s staining were identified by two experienced cytopathologists.

#### MALBAC single-cell amplification

Before single-cell sequencing, we first use MALBAC single-cell amplification, and its experimental steps are as follows: (1) Isolation of the pre-amplification sample preparation area: Before amplification, the preparation process of pre-amplified samples needs to be completed in a separate isolated laboratory or dedicated work area, and special experimental materials and instruments are prepared; the DNA amplification product is stored separately from the pre-amplification reagent; (2) Control group DNA samples (5 μL) are prepared, including positive and negative controls; (3) Placing the cell lysate, pre-amplification enzyme, and amplification enzyme in an ice bath before use, and the other components should be thawed on ice before use; (4) Before the reaction, perform short centrifugation to ensure that the liquid in the reaction system is mixed evenly.

#### Single-cell sequencing

We use the Illumina platform for sequencing, its experimental steps are as follows:

(1) The oligonucleotide is a primer, and the library fragment is a template for DNA replication; (2) After the copy is completed, the library fragments are washed away, leaving the surface of the flow cell as DNA strands complementary to the library template; (3) Because the other end of the single-stranded DNA is a different joint sequence, it can bind to another adjacent oligonucleotide complementarily, followed by “bridge” amplification; (4) After 25–28 cycles are completed, the original single nucleotide sequence scattered on the surface becomes a scattered DNA cluster; (5) Dechain linearization again, cutting and washing the DNA strands on P5, leaving only the DNA strands on P; (6) Add the sequencing primer Read1 SP and the modified DNA polymerase to start DNA replication at the 3′ end of the sequencing primer; (7) To ensure the accuracy of sequencing, synchronous replication of each strand of a site DNA cluster is required; (8) Increase the sequencing length and perform sequencing in the other direction, that is, double-end sequencing.

### Model development preprocess

Each patient in the dataset had MR scans of four sequences: T1C, T1, T2, T2 flair, and tumor mask, along with classification labels.

The goal of this network is to segment three regions of glioma, including enhanced tumor area (ET), non-enhanced tumor area (NET), and edema area (ED); The tumor’s location, size, and classification information obtained from the target detection network were combined with the CTC numerical feature to differentiate the histological type and molecular type.

Four different experienced neurosurgeons, who also received guidance from a senior neuroradiologist (>10 years of clinical experience), applied Segmentation and Segment Editor modules to manually segment the original MRI dataset. All scans have been co-registered to the same anatomical reference using a rigid transform method on the 3D-slicer registration module. Skulls of the brain MRI were removed by 3D-slicer software that can eliminate the influence of skull areas with high-intensity signals. MR scans often display intensity non-uniformities due to variations in the magnetic field. Therefore, N4 bias field correction was used before the image input. We use data augmentation to increase the size of the dataset. To prevent over-fitting in training, data augmentation was used to expand the dataset.

The data augmentation methods used are as follows: (1) Adding random noise to the voxel, the noise worth ranged between −0.1 times and 0.1 times the standard deviation of the voxel. (2) Image size was changed to the original 0.9–0.1 times. (3) Random rotation and translation, with rotation ranging from −20 to +20°, and translation ranging from −30 to +30 and −15 to +15, respectively. (4) Random flip.

According to the 2021 WHO classification criteria, the molecular types of glioma were divided into 11 subcategories, and the histological type was divided into three grades. The grades, segmentations, and subcategories labels of each patient were one-hot encoded. In the preprocessing step, anchor boxes of the tumors were automatically generated according to the segmentation labels. The anchor box was the ground truth of the target detection task.

### Radiomic model development methods

The research of Choi et al. (2021) indicated that the location and shape characteristics of tumors have an important relationship with the IDH classification of gliomas. After adding six location numerical features, 13 shape numerical features, and one age numerical feature, the classification accuracy of IDH was improved by 1.6–5.3 percent compared with the traditional resnet classification model on the three different datasets (Choi et al., 2021). Inspired by that, we designed a model that fuses tumor localization features and shape features for classification. Compared with the radiomics method used in Choi’s et al. (2021) work to obtain tumor location and shape information, obtaining these features directly through the target detection network does not require additional manual operations, which is significantly more time-saving and convenient.

Our model consisted of a segmentation network, target detection network, and classification network. The outputs of the target detection network were first processed and then combined with the numerical information of the CTC to input the classification network to classify the images.

**Figure 2:**
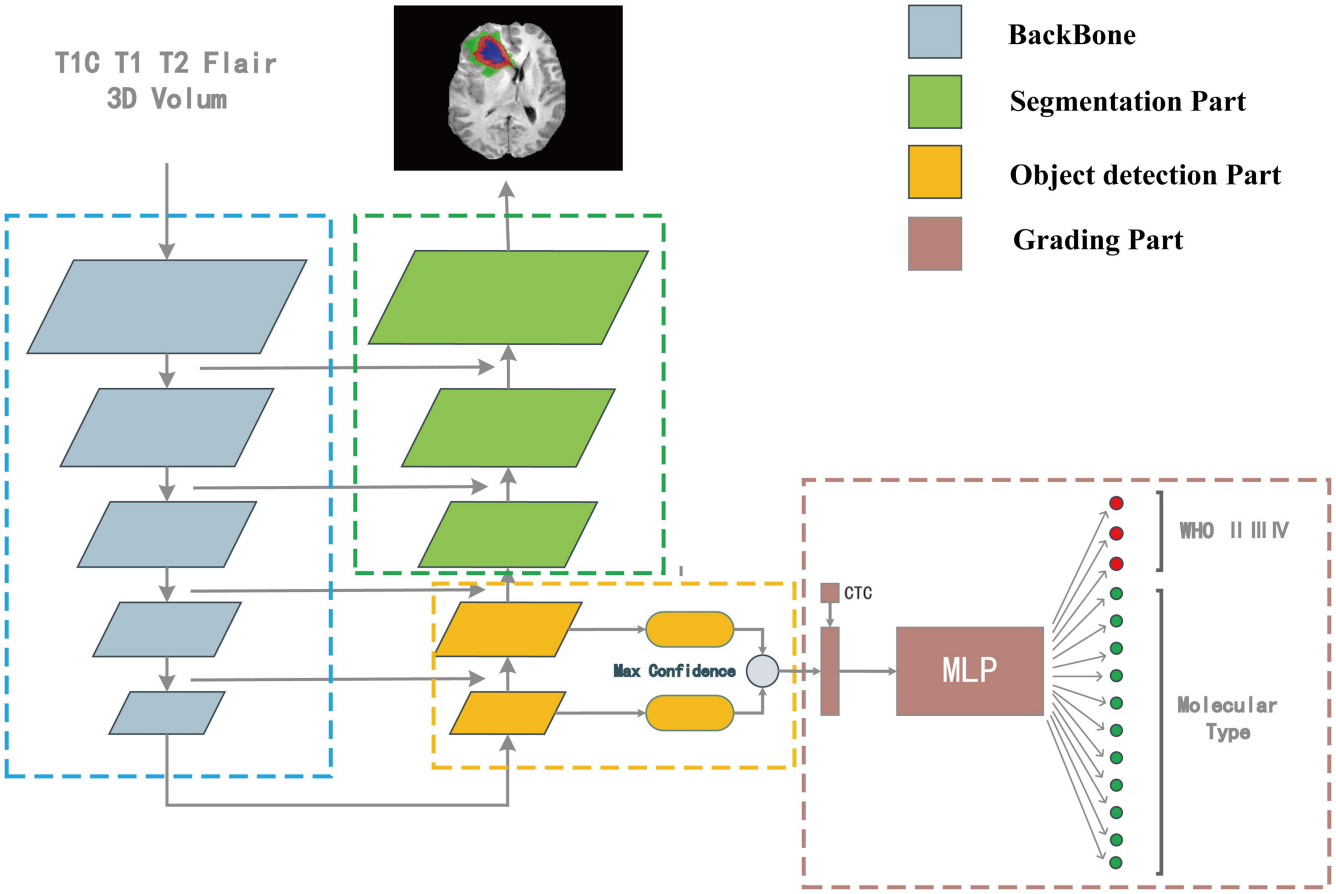
The “integrated diagnosis” model structure. The model is composed of four parts. Features are extracted by backbone first, in the up-sampling process, the object detection part is responsible for extracting the bounding box and classification features, and the segmentation part is responsible for segmenting the tumor region. The output of target detection is connected with the CTC numerical feature as the input of MLP for classification.

Our model structure was divided into four parts: backbone, the segmentation part, target detection part, and classification part. The model’s overall structure is shown in Figure 2. The target detection part was responsible for detecting the location, size, and classification information from the features which were extracted by the backbone. The part between the detection head and the backbone network was called the Neck, and the Neck part used the FPN which performed several upsampling after the end of the feature extraction section (Lin et al., 2017). The outputs of the upsampling layers were input into the detection heads to detect multiscale tumors. The position, size, and classification information of the detection box with the highest confidence were connected with the numerical features of the CTC to co-input a multi-layer perceptron (MLP) for classification. The upsampling part of the target detection network was extended until the feature map of the same size as the input image is obtained. In this process, the high-resolution feature map from the feature extraction part is also received to fuse the high-resolution information and the high-semantic information to form the segmentation part. The structure of the overall segmentation part is similar to the commonly used medical image segmentation network UNet (Ronneberger et al., 2015).

### Backbone

**Figure 3:**
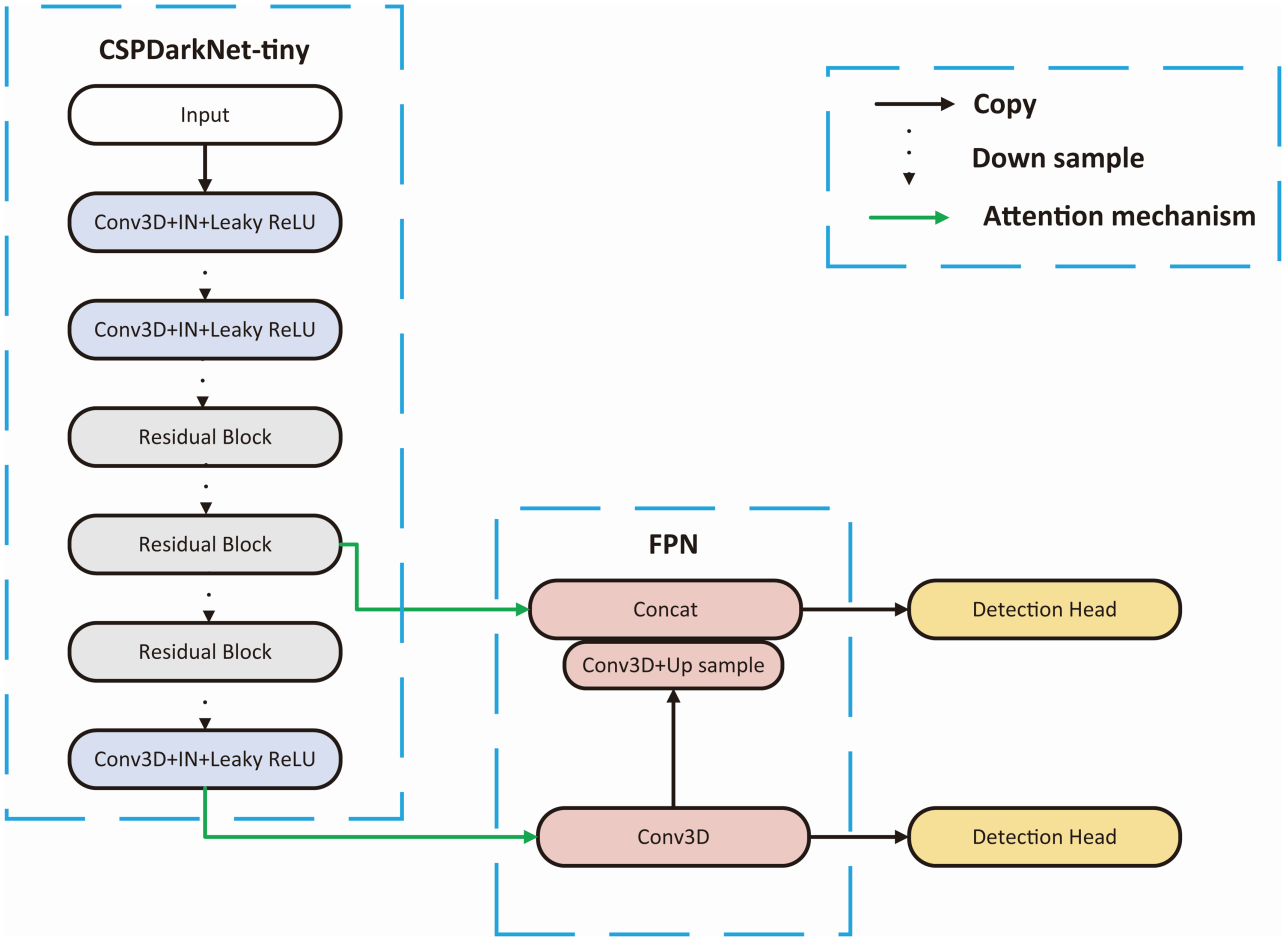
Backbone structure. CSPDarkNet-tiny is used as the backbone for feature extraction, connecting the upper sampling part through the attention mechanism at the first and third layer from the last.

We choose a tiny Cross Stage Partial Network (CSPDarkNet-tiny) as the backbone of the network, which is used to extract useful features in the input images, and the structure of the backbone is shown in Figure 3. The CSPDarkNet-tiny was improved from the DarkNet53-tiny network, inspired by the work of CSPNet (Wang et al., 2020). CSPNet solves the problem of large computation in inference from the perspective of network structure design. The feature extraction part mainly used two kinds of layers: 3-dimensional Convolution + Instance Normalization + Leaky ReLU (CIL) and residual block (Ulyanov et al., 2016). The batch size in our experiment is usually only 1–2, and using batch normalization will lead to model instability (Ioffe and Szegedy, 2015), so instance normalization was used in our network (Wu and He, 2018). The feature extraction section completed 32 x downsampling to facilitate adequate feature extraction. Moreover, the number of convolution convolutions in each stage of the feature extraction part is different, and each residual block contains three convolution operations, mainly because some studies indicate that the model performs better when the deep network blocks (convolution + normalization + activation) have much number of layers (He et al., 2016; Woo et al., 2018; Liu et al., 2022).

### Segmentation part

The segmentation network was formed by extending the upsampled part of object detection until the obtained feature map has the same size as the input feature. The overall network structure was U-shaped, and the segmentation head divided the tumor into four regions: ET, NET, ED, and background.

### Target detection part

**Figure 4:**
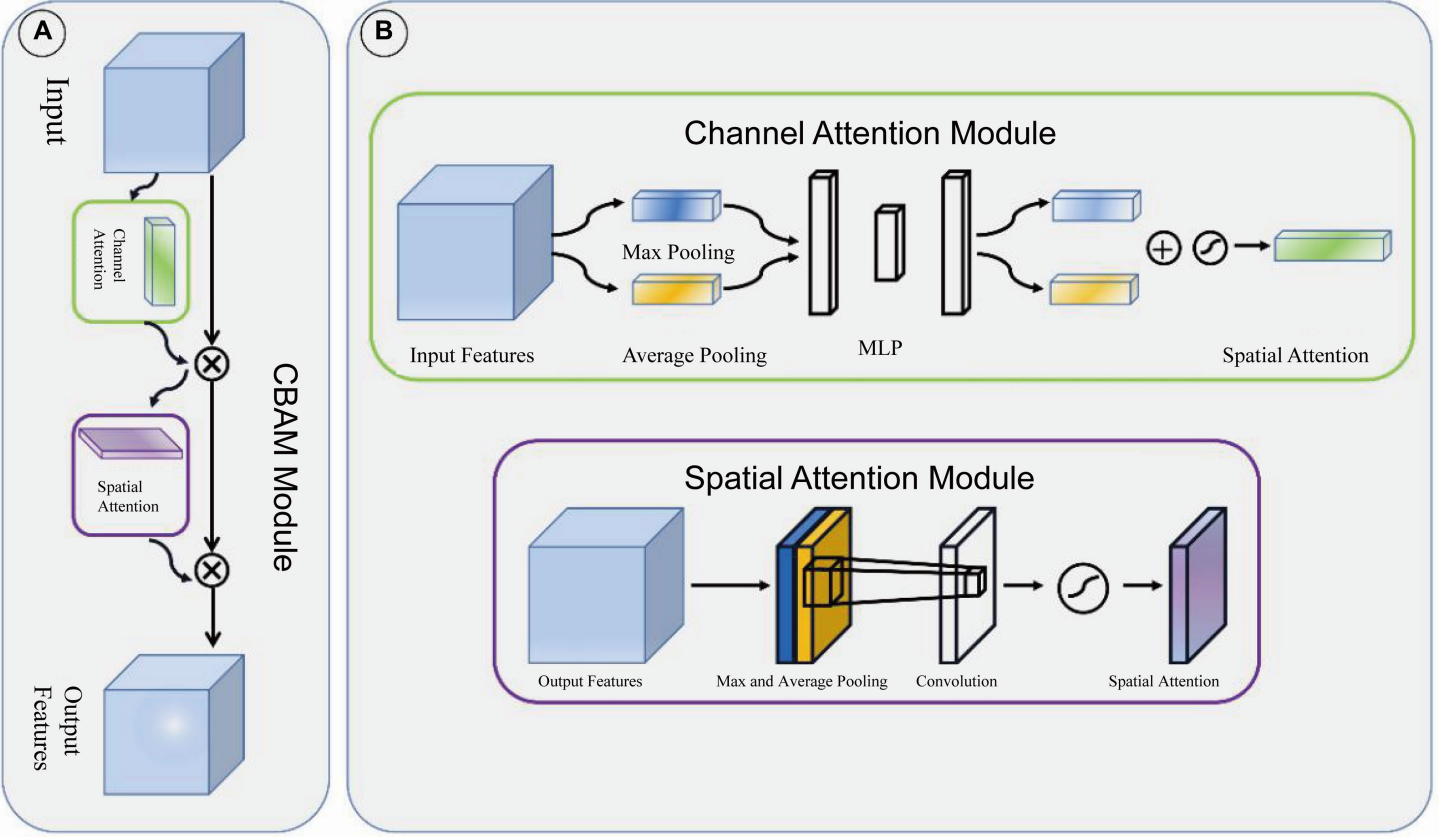
CBAM module. **(A)** CBAM module is consist of channel attention module and spatial attention module. **(B)** The structure of channel attention module and spatial attention module.

Adjacent feature maps from the bottom-up and top-down were concatenated together on the channel and then input to the detection head. Therefore, the input of the head contained rich spatial information from the bottom-up and rich semantic information from the top down. As shown in Figure 3, the Neck part mainly adopts the FPN structure. In addition, to better integrate the features from the backbone network, we also used the CBAM module, whose specific structure is shown in Figure 4. The output of the head contains a lot of bounding boxes with confidence features, position features, size features, and classification features of the bounding boxes. The confidence represents the Intersection over Union (IOU) between the prediction box and the ground truth box. The features of the prediction box with the largest confidence were extracted as the input of the classification network.

### Classification part

**Figure 5:**
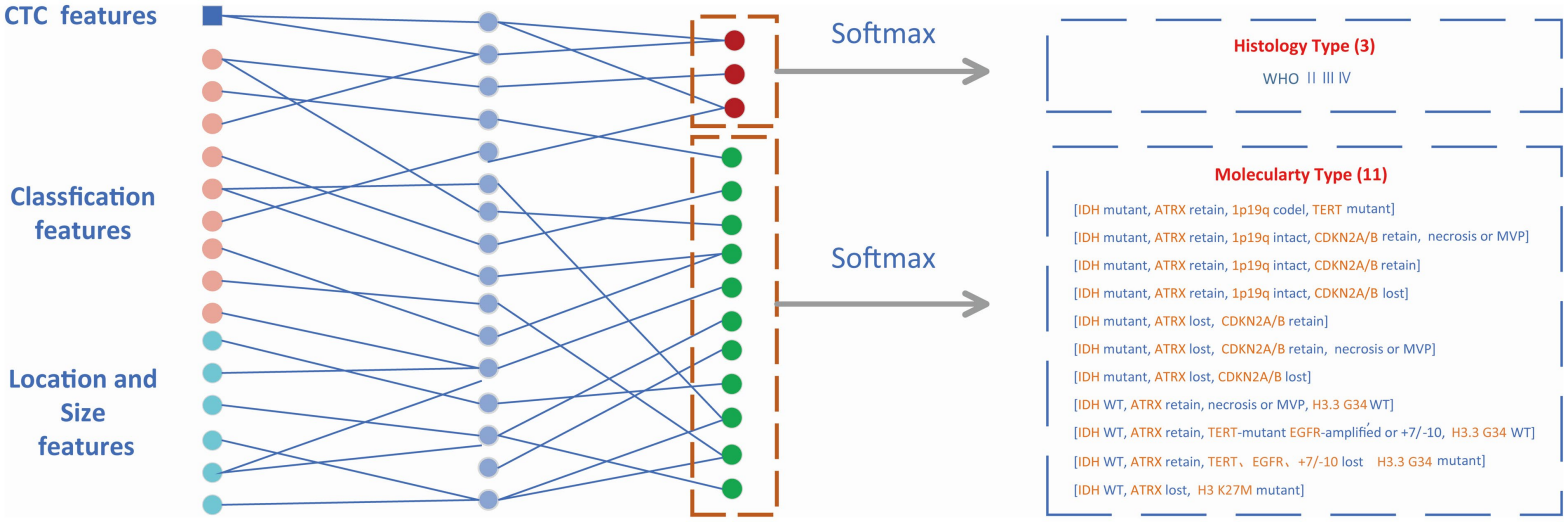
Classification module. Non-invasive prediction of Histology type and Molecular type of gliomas.

The features from the target detection network contained 14 classification features (WHO 3, molecular type 11) and 6 box features (location 3, size 3). The location and size of the prediction box represented the location and size of the glioma to some extent. These numerical features were combined with CTC numerical features and then input into the MLP module which is shown in Figure 5. The outputs of MLP were input into the softmax layer to obtain the final classification of WHO grades and molecular subtypes.

### Loss function

The loss function consists of four parts: coordinate loss, confidence loss, classification loss, and segmentation loss. The confidence loss and regression loss in the object detection part refer to the YOLOv4 model (Bochkovskiy et al., 2020). Cross entropy loss was used as classification loss and segmentation loss.

#### Coordinate loss


$$L_{coord}=\lambda_{\mathrm{coord}}\sum_{i=0}^{H\times W\times D}\sum_{j=0}^{M}I_{ij}^{obj}\left(2-w_{i}\times h_{i}\right)\left(1-DIOU\right)$$

$\lambda_{\mathrm{coord}}$ represents the weight coefficient of coordinate loss, $\sum_{i=0}^{H\times W\times D}\sum_{j=0}^{M}$ indicates traversing all prediction boxes, H,W , and D are the sizes of feature maps, M is the number of anchor boxes at each grid cell on the feature map, $I_{ij}^{obj}\;$ represents whether it is a positive sample (the IOU of the prediction box and ground truth box is >0.5 and is the largest), which is either 0 or 1. The specific expression of DIOU is shown in Equation (2):


$$\begin{array}{c} \mathrm{DIoU}=\mathrm{IoU}-\frac{\rho^{2}\left({\mathrm{b,b}}^{\mathrm{gt}}\right)}{c^{2}} \end{array}$$

Where, $\;\rho^{2}\left(b,b^{gt}\right)$ represents the Euclidean distance between the center points of the prediction box and the ground truth box, and c represents the Euclidean distance that can contain the center points of both the prediction box and the ground truth box (Zheng et al., 2019).

#### Confidence loss


$$\begin{array}{c} L_{\mathrm{conf}}=\lambda_{\mathrm{conf}}\left[-\sum_{i=0}^{K\times K}\sum_{j=0}^{M}I_{\mathrm{ij}}^{\mathrm{obj}}\log\left(C_{i}\right)-\sum_{i=0}^{K\times K}\sum_{j=0}^{M}I_{\mathrm{ij}}^{\mathrm{noobj}}\log\left(1-C_{i}\right)\right] \end{array}$$

The two terms equation refer to positive sample confidence loss and negative sample confidence loss, respectively. $I_{\mathrm{ij}}^{\mathrm{noobj}}$ represents the negative sample, whose IOU of the prediction box and label box is <0.1. $\;\;\lambda_{\mathrm{conf}}$ represents the weight coefficient of confidence loss.

#### Classification loss


$$\begin{array}{c} \begin{array}{c} L_{\mathrm{cls}}=-\lambda_{\mathrm{cls}}\sum_{C\in \mathrm{types}}\sum_{c\in \mathrm{classes}\;}\\ \left[{\hat{p}}_{i}\left(c\right)\log\left(p_{i}\left(c\right)\right)+\left(1-{\hat{p}}_{i}\left(c\right)\right)\log\left(1-p_{i}\left(c\right)\right)\right] \end{array} \end{array}$$

Where, ${\hat{p}}_{i}\left(c\right)$ represents the prediction class, $\;p_{i}\left(c\right)$ represents the true class, C represents the types of classification (WHO, IDH, ATRX, or 1p19q), and c represents classes in each classification. $\lambda_{\mathrm{cls}}$ represents the weight coefficient of classification loss.

#### Segmentation loss


$$\begin{array}{c} L_{seg}=-\lambda_{seg}\sum_{c=1}^{C_{seg}}\log\frac{\exp\left(x\right)}{\sum_{C_{seg}}^{i=1}\exp\left(x\right)}y \end{array}$$

x is the output of the segmentation head, y is the one-hot code of the segmentation label, $C_{seg}$ represents the number of classes that need to be segmented, and $\lambda_{seg}$ represents the segmentation loss.

The total loss function is the sum of the four loss functions, as shown in equation


$$\begin{array}{c} L_{\mathrm{object}}=L_{\mathrm{coord}}+L_{\mathrm{conf}}+L_{\mathrm{cls}}+L_{\mathrm{seg}} \end{array}$$

### Evaluation index

#### Classification index

The classification indexes used in this paper include accuracy, precision, and recall, which will be introduced one by one in the following, and the confusion matrix is used to calculate the classification metrics (Stehman, 1997).

**Accuracy:** The accuracy represents the proportion of correctly predicted samples in the total sample, as shown in the equation:


$$\begin{array}{c} Accuracy=\frac{TP+TN}{TP+FN+FP+TN} \end{array}$$

**Precision:** Precision represents the proportion of truly positive samples among the samples predicted to be positive by the model, as shown in the equation:


$$\begin{array}{c} Precision=\frac{TP}{TP+FP} \end{array}$$

**Recall:** For all positive samples, Recall represents the proportion of actual positive samples that are predicted to be positive, as shown in the equation:


$$\begin{array}{c} Recall=\frac{TP}{TP+FN} \end{array}$$

#### Target detection index

**Intersection over union (IOU):** $A_{a}$ represents the predicted bounding box, $A_{m}$ represents the true bounding box. IOU is shown in equation:


$$\begin{array}{c} IOU=\frac{A_{a}\cap A_{m}}{A_{a}\cup A_{m}} \end{array}$$

#### Segmentation index

**Dice:** Pred is the predicted tumor area, and true is the real tumor area. Dice is shown in equation (Milletari et al., 2016):


$$Dice=\frac{2*\left(pred\cap true\right)}{pred\cup true}$$

### Learning curve during the process of model training

**Figure 6:**
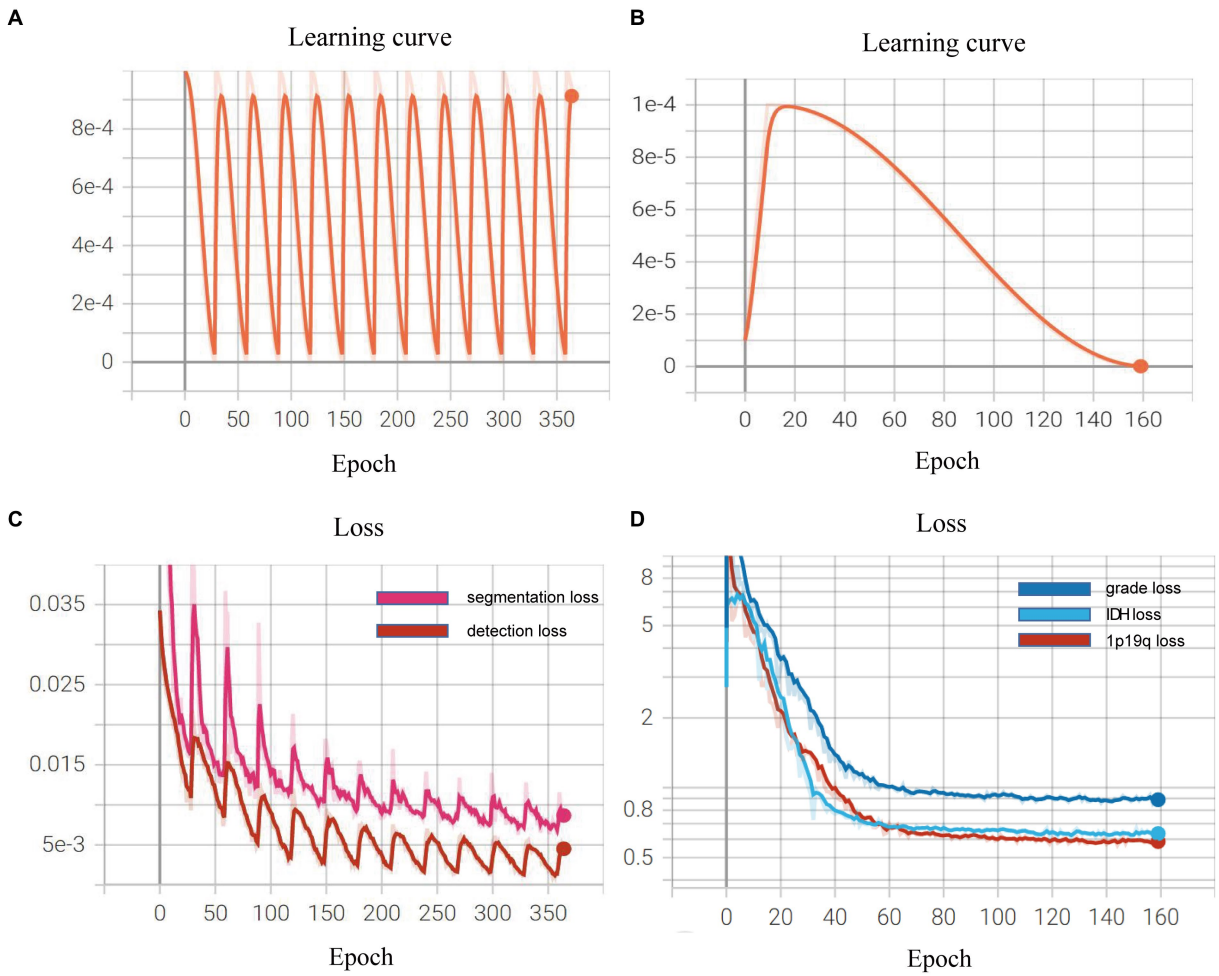
Learning curves during the model training: **(A,B)** show the learning rate and loss function value curves in this stage; **(C,D)** show the learning rate and three classification loss value curves.

**Figure 7:**
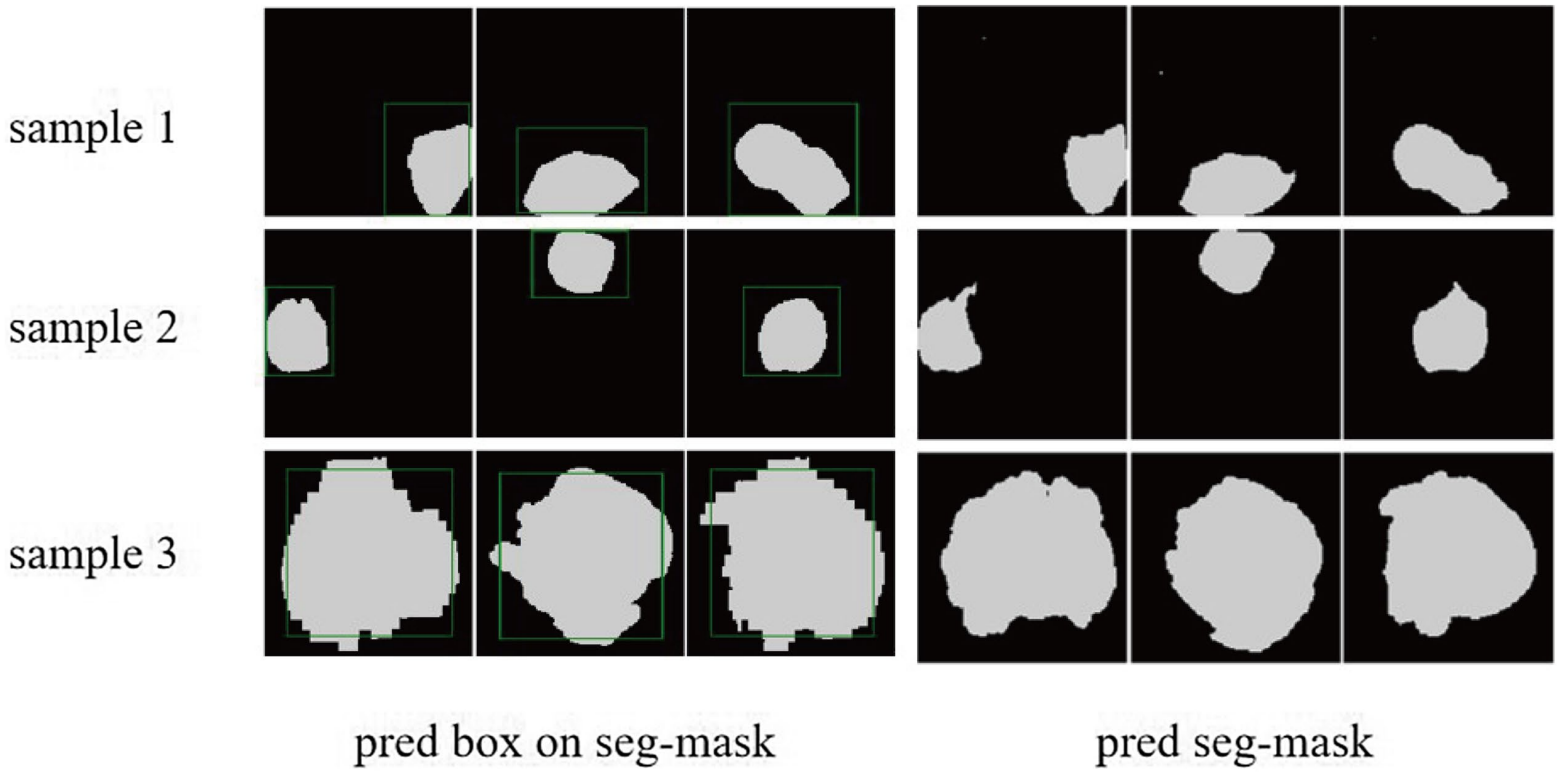
The cases of tumor area detection and segmentation via the proposed model. The three rows from top to bottom are the three views of three glioma samples, respectively. The left side is the prediction box obtained from the object detection part (green line) and the true mask of glioma segmentation (white), and the right side is the segmentation result predicted by the segmentation module.

We demonstrated the learning curves that show the training loss during the process of model training. First, the backbone, target detection, and segmentation parts are trained with the cosine annealing learning rate decay strategy. The loss value decreases steadily with the increase of the learning rate decay period. Figures 6A,B of the revised manuscript show the learning rate and loss function value curves in this stage. Then, the classification part was trained, which adopted the learning rate decay strategy of first warming up in 10 epochs and then declining in 150 epochs. The learning rate and three classification loss value curves are shown in Figures 6C,D. Figure 7 illustrates the cases of tumor area detection and segmentation via the proposed model in this study.

## Discussion

Preoperative and non-invasive diagnosis of the molecular subtype is very critical for glioma treatment. The combined methods of radiomics and LB have great potential to non-invasively diagnose glioma grading and molecular markers since they are both easy to perform. This is the first study that combing these two technologies for glioma diagnosis.

In this study, we apply multimodal MRI to construct a multi-task DL model, which the backbone of its network architecture is built by CSPNet. CSPNet solves the problem of large computation in inference from the perspective of network structure design (Wang et al., 2020). The authors of CSPNet argue that the problem of excessive inference computation is due to the duplication of gradient information in network optimization. Therefore, CSP is used to first divide the feature map of the basic layer into two parts, and then merge them through the cross-stage hierarchy, which can reduce the calculation amount while ensuring accuracy. In this protocol, a relatively lightweight DarkNet-tiny will be selected as the basis to fuse the CSP connection to form the final CSPDarkNet-tiny structure. In terms of algorithms, they are different from previous studies because most of them are built based on CNN purely (Shaver et al., 2019; Matsui et al., 2020; Choi et al., 2021; van der Voort et al., 2022).

To date, with the in-depth study of LB, CTC as an important branch has been applied to the non-invasive diagnosis of glioma. In our previous study (Qi et al., 2021), it has been confirmed that peripheral blood CTC is related to IDH mutant. However, the relationship between CTC and other important molecular targets has not been further explored, and we have not found other similar studies. In this study, we combine CTC count as a very important parameter with the DL model to explore its value in molecular and graded noninvasive diagnosis of glioma, which is unprecedented in the current study. However, it must be clarified that some glioma subtypes are assigned to fewer training samples and cannot rely on the model for subtype diagnosis. Therefore, we further proposed CTC sequencing of individual molecular targets as the basis for the diagnosis of glioma subtypes. Since the inherent black-box nature of deep learning models and less transparency, we will add relative modules to improve the understandability of existing models based on the premise of the stable prediction performance of the models in the future (Dasanayaka et al., 2022).

Since the publication of the WHO-CNS classification guidelines in 2021, scholars around the world have been committed to the application of radiomics or LB techniques to achieve “precisely integrated prediction” of gliomas before surgery, since the noninvasive prediction of important molecular markers is essential to guide the treatment of patients who are inoperable or have tissue biopsy. Simply relying on radiomics features to find the correlation with the molecular pathological features of gliomas can no longer meet the need for an accurate diagnosis of gliomas. CTC features are a promising biomarker that may provide new directions in the exploration of “precision diagnosis” based on the radiomics. How to combine the preoperative radiomics features, CTC features or other LB markers of glioma patients is the direction that requires the joint efforts of global scholars in the future, At the same time, it is necessary to provide matched molecular marker information of glioma patients to ensure the feasibility of the study.

We firmly believe that this innovative work will surely lay a good foundation for the “precisely integrated prediction” of glioma and shed a new direction for future research.

## Ethics statement

This study is conducted in accordance with the Declaration of Helsinki and the Code of Ethics for Human Medicine and Health Research and has been approved by the Ethics Committees of The Second Affiliated Hospital of Nanchang University (IIT-D-2022-003), Renmin Hospital of Wuhan University (WDRM2021-K109), and registered at ClinicalTrails.gov on September 10, 2022, with Identifier NCT05536024. All recruited patients signed informed consent.

## Author contributions

PH, YZQ, TFY, LGY, QXC, and XGZ: study design investigators. LX, DLY, SW, XL, PPX, RY, DFW, and DZ: deep learning algorithm design. YZQ, TFY, LGY, QS, XYZ, SHD, SWL, JC, and GD: CTC collection and analysis. PH, LX, YZQ, YW, QS, YX, SLD, and YY: literature search. PH, LX, YZQ, TFY, QXC, and XGZ: protocol preparation, editing, and review. All authors contributed to the article and approved the submitted version.

## Funding

This study is supported by the National Natural Science Foundation of China (nos. 81960456 and 82172989 to XGZ; 82072764 to QXC) plays a role in supporting data collection, data processing redefined molecular type according to the 2021 WHO-CNS classification; no. 82201515 to JC, Wuhan University Teaching Reform Project (no. 2022ZG188 to GD), and Cross-innovation talent project of Renmin Hospital of Wuhan University (JCRCWL-2022-005 to QXC and DZ) play a role in supporting data collection, data processing, and deep learning radiomic model development; Postdoctoral Research Foundation of China (nos. 2022M712464 to YZQ and 2022M721452 to TFY), Natural Science Foundation of Jiangxi Province Youth Project (no. 20224BAB216074 to TFY), Key Research and Development projects in Jiangxi (20212BBG71012), and Jiangxi Branch of National Clinical Research Center for Geriatric Diseases (2021ZDG02001) play a role in supporting CTC enrichment, single-cell sequencing.

## Conflict of interest

The authors declare that the research was conducted in the absence of any commercial or financial relationships that could be construed as a potential conflict of interest.

## Publisher’s note

All claims expressed in this article are solely those of the authors and do not necessarily represent those of their affiliated organizations, or those of the publisher, the editors and the reviewers. Any product that may be evaluated in this article, or claim that may be made by its manufacturer, is not guaranteed or endorsed by the publisher.
